# Recent Developments at DG Competition: 2017/2018

**DOI:** 10.1007/s11151-018-9671-7

**Published:** 2018-11-01

**Authors:** Andrea Amelio, Thomas Buettner, Cyril Hariton, Gábor Koltay, Penelope Papandropoulos, Geza Sapi, Tommaso Valletti, Hans Zenger

**Affiliations:** 1 Directorate-General for Competition, European Commission, MADO 18/058, 1049 Brussels, Belgium; 2 Directorate-General for Competition, European Commission, MADO 17/025, 1049 Brussels, Belgium; 3 Directorate-General for Competition, European Commission, MADO 17/037, 1049 Brussels, Belgium; 4 Directorate-General for Competition, European Commission, MADO 17/021, 1049 Brussels, Belgium; 5 Directorate-General for Competition, European Commission, MADO 17/053, 1049 Brussels, Belgium; 6 Directorate-General for Competition, European Commission, MADO 17/005, 1049 Brussels, Belgium; 7 Imperial College Business School, South Kensington Campus, London, SW7 2AZ UK; 8 Directorate-General for Competition, European Commission, MADO 17/026, 1049 Brussels, Belgium; 9 Directorate-General for Competition, European Commission, MADO 17/014, 1049 Brussels, Belgium

**Keywords:** Competition policy, Antitrust, Mergers, State aid, Counterfactual, Google, Price discrimination

## Abstract

The Directorate General for Competition at the European Commission enforces competition law in the areas of antitrust, merger control, and state aids. This year’s article provides first a general presentation of the role of the Chief Competition Economist’s team and surveys some of the main achievements of the Directorate General for Competition over 2017/2018. The article then reviews: the Google Search (Shopping) case, the role of price discrimination in state aid cases; and the use of counterfactuals in merger cases where alternative transactions might have occurred absent the merger.

This article provides first an overview of the activity of the Directorate General for Competition of the European Commission (DG Competition) in 2017/2018 that is related to antitrust, mergers and state aid (Sect. 1). In the following sections, the contribution by the Chief Economist Team (CET) to the economic analysis in specific cases is presented. In particular, Sect. 2 reviews the Google Search (Shopping) antitrust decision; Sect. 3 discusses state aid cases where the CET worked on identifying the circumstances when subsidies in the form of differential pricing can be considered distortive of competition; and, finally, Sect. 4 elaborates on the analysis of counterfactuals in recent mergers that involved the possibility of alternative transactions.

## Main Developments in 2017/2018

### The Chief Competition Economist Team

The CET is a part of DG Competition. Its staff consists of 30 economists (mostly holding PhDs) with a mix of permanent and temporary positions. The CET is headed by the Chief Competition Economist, who is an external academic who is recruited for a three-year term.

The CET has both a support role and a scrutiny role. As part of its support role, the team provides guidance on methodological issues of economics and econometrics in the application of EU competition rules. It contributes to individual competition cases—in particular, the ones that involve complex economic issues and quantitative analysis—and to the development of general policy instruments, as well as assisting with cases that are pending before the Community Courts.

Members from the CET who are assigned to specific cases have a specific and independent status within case teams, and report directly to the Chief Competition Economist. As part of the scrutiny role, the Chief Competition Economist can report his opinion directly to the Director-General of DG Competition as well as to the Competition Commissioner, providing her with an independent opinion on the economic aspects of a case before she proposes a final decision to the European Commission.

The CET is active in DG Competition’s three main areas of policy: antitrust, merger control, and state aid. Historically, the CET’s main domain of activity is merger investigations—typically 50–60% of CET’s time—while CET’s work on state aid and antitrust is typically in a range of 20–25% of the CET’s time each.

### DG Competition’s Activities in 2017/2018^1^

#### Antitrust

Between January 2017 and July 2018, the Commission took decisions in seven (non-cartel) antitrust cases: E-book MFNs and related matters (Amazon)^2^ in May 2017; Google Search (Shopping)^3^ in June 2017; Baltic rail transport^4^ in October 2017; International Skating Union’s eligibility rules^5^ in December 2017; Qualcomm exclusivity^6^ in January 2018; Greek lignite^7^ in April 2018; Upstream gas supplies in Central and Eastern Europe (Gazprom)^8^ in May 2018; Asus, Denon & Marantz, Philips and Pioneer^9^; and Google Android^10^ in July 2018. The CET has predominantly been active in the Amazon case, which was discussed in this journal last year, in the Google Search (Shopping) case, which is discussed in Sect. 2 below, and in the Qualcomm exclusivity and Google Android cases.

With respect to court decisions, the Court of Justice of the European Union delivered in September 2017 its judgment on Intel’s appeal of the European General Court’s judgment that confirmed the Commission’s 2009 decision against Intel.^11^ The Court of Justice referred the case back to the General Court; the Court of Justice directed that, as the “as efficient competitor test” played an important role in the Commission’s assessment, the General Court was required to examine all of Intel’s arguments concerning that test, which the General Court had failed to do.

The Court of Justice also clarified a number of questions that are related to the assessment of excessive prices. In a preliminary ruling in Latvijas Autoru apvienība published in September 2017,^12^ the Court held that for the purposes of examining whether a copyright management organisation applies “unfair prices”, it is appropriate and sufficient to compare its rates with those either applicable in neighbouring Member States or in other Member States adjusted in accordance with the purchasing power parity index—provided that the selection of these Member States follow objective, appropriate, and verifiable criteria and that the comparisons are made on a consistent basis.

#### Mergers

During the period January 2017 to July 2018, 612 merger transactions were notified to DG Competition.^13^ A significant share of these cases (447) were so-called “simplified” cases.^14^ Out of the 165 remaining cases, 105 were cleared after a “first-phase” investigation, with remedies in 25 of these cases.

When remedies at the end of the first-phase investigation are not adequate to resolve the competitive concerns that are identified at that stage, or if none are submitted, the transaction is subject to a “second-phase” or “in-depth” investigation.^15^ When competition concerns are confirmed during this phase, a Statement of Objections is issued to inform the Parties to the transaction of the Commission’s preliminary conclusions. During the 2017–2018 period, six mergers were authorized subject to remedies after an in-depth investigation,^16^ and one was authorized without remedies,^17^ whilst two cases were prohibited and three were abandoned during the in-depth investigation.^18^^,^ 19

The CET was involved in all of these second-phase investigations as well as in some complex first-phase investigations, with a focus on specific topics such as innovation competition and the analysis of patent data,^20^ common shareholding,^21^ non-horizontal effects due to bundling, horizontal effects in seemingly vertical transactions that involve integrated firms, and counterfactuals that involve alternative transactions. This last topic is developed in Sect. 4.

In terms of court developments, in March 2017 the General Court annulled on procedural grounds the Commission’s 2013 decision that prohibited the acquisition by UPS of TNT Express.^22^ The Court held that the Commission had not communicated to UPS the final version of an econometric model that was used in the contested decision, thus infringing UPS’ rights of defence. The Commission has appealed the annulment before the Court of Justice, and the appeal procedure is on-going.^23^

#### State Aid

Between January 2017 and July 2018, the Commission took 444 decisions in the area of state aid, most of which concluded that the actions were compatible with the Commission’s criteria for justifiable actions or did not actually involve aid.^24^ After the Apple recovery decision to Ireland in August 2016,^25^ the Commission continued its investigation of the tax ruling practices of Member States and adopted two further negative decisions that concerned tax rulings in Luxembourg: Amazon in October 2017^26^ and Engie in June 2018.^27^ In the Amazon case, the Commission concluded that the tax ruling endorsed an unjustified method to calculate Amazon’s taxable profits in Luxembourg. In particular, the level of the royalty payment from the operating company to the holding company was artificially inflated and did not reflect the economic reality of the tasks that were performed by each entity.

The CET has been mainly involved in cases that are related to transport (airports, motorway concessions, railway companies), energy (capacity mechanisms),^28^ regional aid, and banks. In the transport sector, the CET worked with financial models and business plans to assess a wide range of issues, such as the valuation of concession extensions or assessments of the least distortive conditions for concession extensions. The CET also contributed to calculating proportionate amounts of investment or operating aid to regional airports. For “services of general economic interest” (SGEI)—e.g., in the postal or transport sectors—the CET worked on the calculation of proportionate compensations for the delivery of “universal” or “public service obligation” (PSO) contracts.

With respect to court decisions, two notable state aid judgments can be mentioned. In the Charleroi Airport case, the General Court confirmed the method to calculate the benchmark concession fee for the management of the airport against which to compare the concession fee actually paid by the airport manager.^29^ In the Hinkley Point C case, the General Court upheld the Commission’s analysis with regard to the conformity of the aid for the construction of a new nuclear power plant in the UK (subject to commitments by the UK).^30^ The subsidies were mostly in the form of feed-in tariffs^31^ and a government guarantee on future bond issuance (the CET contributed to establishing market-based pricing for the guarantee).

Section 3 discusses two recent decisions where the CET worked on identifying the circumstances when subsidies in the form of differential pricing can be considered distortive of competition.

## Google Search (Shopping)

The Commission is currently pursuing three cases against Google, normally referred to as: Shopping, Android and AdSense. On 27 June 2017, the Commission decided that in the Shopping case Google abused its dominant position in 13 national markets^32^ for general search services in the European Economic Area (“EEA”), in breach of Article 102 TFEU and Article 54 of the EEA Agreement. The abusive conduct is defined as “*the more favourable positioning and display, in Google’s general search results pages, of Google’s own comparison shopping service compared to competing comparison shopping services*”. The Decision requires Google to stop the illegal conduct within 90 days. It foresees penalty payments in the event of non-compliance, and it imposes a fine of €2.4 billion on Google that takes account of the duration and gravity of the infringement. Google has appealed the decision.

Comparison shopping services allow consumers to search for products online, compare their characteristics and prices, and provide links to the webpages of online retailers. Therefore, comparison shopping services act as intermediaries between consumers and online retailers. Examples of companies that offer comparison shopping services include Idealo, Twenga, Kelkoo, and Pricerunner, among many others. Since 2004, Google has offered its own comparison shopping service. These companies have contractual relationships with retailers. Retailers provide standardised data that allow consumers to compare the products, and the retailers typically pay a pay-per-click fee for the comparison shopping services. Consumers do not pay for the comparison shopping services.

Consumers access comparison shopping services either directly through the webpages of the companies, or indirectly through other webpages that display them. The Decision explains that Search engines—especially Google Search—are an important source of traffic for comparison shopping services. Google is dominant in the market for general search services in all EEA Member States, with a market share of over 90% in most Member States.

The Decision concludes that Google positions and displays its own comparison shopping service more favourably, due to the combination of two practices. First, Google applied algorithms which were prone to reduce the ranking of rival comparison shopping services on its general search page. This change in the rankings—which is commonly known as demotions—happened without any change in the design and content of those competing comparison shopping services. Second, the demotion algorithm did not apply to Google’s own comparison shopping service, which instead was systematically given prominent placement and display on its general search page. When a consumer enters a relevant query into Google general search engine, the results of Google’s comparison shopping service are positioned and displayed at, or near, the top of the general search results in the so-called Shopping Unit with rich graphical features.

The Decision does not object to either of these elements of the conduct on their own, but rather that these elements are not applied to Google’s comparison shopping service and competitors in the same way. It also does not object to Google’s applying certain relevance standards for ranking its general search results; it objects that these relevance standards are different between Google’s comparison shopping service and its competitors. Similarly, the Decision does not object to Google applying rich features and prominent display for certain results; it instead objects that these rich features and prominent display are applied only to Google’s own service and not to competitors’ services.

The Decision concludes that the conduct has potential anti-competitive effects in the market for comparison shopping services: the conduct has the potential to foreclose competitors in this market. As a result, consumers rarely even see—let alone click on—rival comparison shopping services. Consumers’ choices are therefore constrained; and rival comparison shopping services have fewer incentives to innovate, because—irrespective of the quality that they offer—users will generally not find them. According to the Decision, these effects may also lead to higher online retail prices. The Decision also finds that the conduct has the potential to increase Google’s market power in the market for general search.

The Decision argues that Google did not provide verifiable evidence that its conduct is indispensable to the realisation of its claimed efficiencies and that there are no less anti-competitive alternatives to the conduct that are capable of producing the same efficiencies. It also did not provide arguments or evidence to show that the claimed likely efficiencies that are brought about by the conduct outweigh any likely negative effects on competition and consumer welfare in the affected markets.

The remedy requires Google to treat its own comparison shopping service and those of rivals equally. It does not prescribe a specific solution, so long as this principle is adhered to.

There are four interesting economic aspects of the Decision: (1) the source of dominance in general search; (2) the theory of foreclosure; (3) the underlying incentives to foreclose; and (4) evidence of foreclosure. In the following, our discussion focuses on these aspects of the case.

### The Source of Dominance in General Search

The Decision identifies the following fundamental sources of Google’s dominance in the market for general search: the importance of scale, indirect network effects, and brand effects.

Scale is identified as an important factor that increases the barriers to entry of rival search engines. Search engines need to churn large volumes of queries in order quickly to update and refine the relevance of results. The larger is the number of queries that are submitted to and results that are clicked in a search engine, the faster the search engine learns to detect a change in users’ behaviour and adapts and improves the relevance of the results. This holds for both free ‘blue link’ results and for paid search ads.

The importance of scale is further amplified by the fact that queries do not all have the same frequencies of usage. As the Decision explains, there are common queries (“head” queries) and uncommon queries (“tail” queries). Tail queries make it more difficult for a smaller, rival search engine to provide relevant results. The poor performance for the tail queries affects negatively the user experience, and this reduces traffic for the smaller search engine in general. The Decision acknowledges and endorses the fact that a sufficiently large amount of data is a necessary input for the ability to compete in these markets.^33^

Indirect network effects are an important characteristic of two-sided platforms, as is recognised in the economic literature.^34^ Both general search services and comparison shopping services are two-sided platforms. The Decision relies on the economic intuition developed in the literature that there are positive feedback effects between the advertisers on one side of the platform and the users who request search services on the other side. In line with the results of this economic literature, the Decision finds that the higher is the number of users, the higher is the value for advertisers to post (and to bid for) their ads on the page that contains the results of a search.

As a result, for advertisers who typically are faced with fixed costs in developing their ad campaigns, there is little incentive to use other search engines given the expected smaller number of users who are attracted to rival general search services. Similarly, this dynamic is also present in the market for comparison shopping services, where the two groups of customers are potential online consumers and online retailers.

Brand awareness seems also to characterise the behaviour of users who consume general search services. Contrary to Google’s claim, the Decision finds that overall users are faithful to one search engine and that they do not tend to multi-home. The Decision shows evidence that users of general search services were found to use only Google for the majority of their queries, while only occasionally diverting to other search engines.

These effects make Google of significant commercial importance for users who look for or want to be directed to comparison shopping services. In other words, when shopping online and wanting to compare prices, users often search on Google and then, based on Google’s suggestion, to make a choice of where next to go. This finding is consistent with the fact that users typically have limited information and rely heavily on the suggestions of Google. There is evidence that users do not read search results in pages other than the first page and have a very high tendency to click on the results ranked in top slots in the page.^35^

### Theory of Foreclosure

The Google Shopping decision is a leveraging abuse case. Google used its dominance in the market for general search services to give its comparison shopping service an artificial advantage and exclude competing comparison shopping services, which leads to anticompetitive foreclosure.

This approach is similar to that of the Guidance Paper where anticompetitive foreclosure is defined as “*a situation where effective access of actual or potential competition to supplies or markets is hampered or eliminated as a result of the conduct of the dominant undertaking whereby the dominant undertaking is likely to be in a position to increase prices to the detriment of consumers.*”^36^

The anticompetitive foreclosure is established in the Decision by the likelihood of having negative consequences on competition. Ultimately, the foreclosure of competitors would also have negative consequences on users as it could increase advertising costs and thus eventually lead to higher product prices. What is more, from the outset, the reduction of choice could determine the inability of consumers to reach the most relevant shopping opportunity. Also, a concentrated market that results from the foreclosure could reduce the pace of innovation.

The finding of Google’s anticompetitive foreclosure in the market for comparison shopping services is also dependent on the Decision’s assessment of the role of “merchant platforms” (e.g., Amazon, eBay). The Decision reaches two conclusions on merchant platforms. First, it concludes that comparison shopping services and merchant platforms are in different product markets. They serve different purposes for customers, and merchant platforms have business models that are closer to online retailers.^37^ This is also complemented by the evidence that merchant platforms and comparison shopping services serve different groups of online merchants.

Second, it explains that, even if the merchant platforms were to be included in the analysis of effects, the importance of comparison shopping services would still be relevant and thus the coverage of Google’s anticompetitive foreclosure would still be non-negligible.

Google has contested these two findings of the Commission. Google states that, on the one hand, Google is not the only way that users have to reach comparison shopping services and online retailers; and, on the other hand, that alternative mechanisms, such as merchant platforms—including Amazon and eBay—are driving competition in this (broader) sector of directing and enabling users to shop online.^38^

### Incentives to Foreclose

While the Decision does not discuss directly Google’s incentive to foreclose, the structure of Google’s well-known business model—which is also described in the Decision—contains an inherent monetisation problem and allows a detailed discussion of foreclosure incentives.^39^

The strategic decision (and commitment) of Google to distribute free general search services is a core element for Google’s incentive to foreclose in adjacent markets, such as the comparison shopping services market. Only clicks on Google’s ads or Google’s comparison shopping service trigger a payment; clicks on the general search results do not. The free Google general search results generate traffic for a wide range of business that offer paid products and services. However, Google cannot directly monetise this traffic.

This feature of the general search services market makes the classical Chicago School critique not applicable. The Chicago School critique argues that a dominant company should have no incentive to foreclose competitors from adjacent markets because the dominant company could make as much profit (if not more) by appropriately pricing its dominant product/service and extracting all the value because of the “one monopoly profit” theory. Google’s strategic decision not to price general search services puts Google in a situation where the only way for Google to appropriate the value that is created by users’ clicks that lead to rival comparison shopping platforms is to appropriate the clicks of those users by directing them to its own comparison shopping service. Sending users to competing comparison shopping services would amount to a net profit loss and, over time, to an increasing risk of users’ bypassing Google and going directly to the competing comparison shopping services.

The static loss of profits that are derived from users who are directed to competing comparison shopping services provides incentives for Google to foreclose competitors in the comparison shopping services market. This static incentive to foreclose is also complemented by the risk of consumers bypassing Google over time if rival comparison shopping services become too successful. The static incentive is thus also reinforced by the dynamic risk for Google to lose its gatekeeper role for consumers who look for (or want to be directed to) comparison shopping services.

### Evidence on Potential Foreclosure

In order to demonstrate Google’s potential to foreclose competitors, the Decision presents several pieces of empirical evidence. First, it shows that the introduction of specific algorithm changes (in particular the launch of the “Panda” algorithm) led to the demotions of competitors’ ranking.^40^ The Sistrix Visibility Index^41^ indicates a substantial decrease in competitors’ visibility and therefore a deterioration of display after the introduction of the Panda algorithm. The Decision also presents evidence that this reduction in visibility and ranking reduces traffic. Data on clicks by search result rank and positioning show that there is a clear link between visibility, the lay-out of Google’s general search results pages and click-through behaviour: results that are displayed higher and in a more visible format attract significantly more clicks than those that are displayed lower or beyond the first page.

Second, the Decision shows that the evolution of the visibility index of competitors was indeed following a similar trend to the generic search traffic of competitors. Moreover, the non-confidential graphs in the Decision show a trend break in the traffic (clicks) to competitors: a stable increase turned into a stable decrease, although the extent of this break varies across countries. The Decision offers documentary evidence that indicates that comparison shopping services suffered a short-term and long-term traffic decreases due to the introduction of systematic demotions.

Third, beyond the impact on traffic of demotions, the Decision assessed an experiment by Google; this assessment addressed the impact on traffic from the rich and prominent display of Google’s own comparison service. In this “ablation” experiment, Google removed the Shopping Unit from its general result pages for a randomly selected group of users. Comparing the click-through rate in this selected group to the click-through rate of the unchanged display allowed an estimation of the causal effect of the more prominent placement of the Shopping Unit with respect to competitors’ traffic. The Decision concluded that the experimental evidence points to an effect on competitors’ traffic. The Decision illustrates this impact on both the generic and text ad (AdWords) traffic of competitors. The Shopping Unit’s effect on text ad traffic (AdWords) suggests that competitors found it difficult to replace lost free generic search traffic by paid text ad traffic.

Finally, in the same period of time, based on the same two metrics (visibility and clicks), Google’s comparison shopping service experienced a sharp increase in its traffic on a lasting basis. Thus, the Decision reaches the conclusion that Google’s conduct had the effect of promoting Google’s comparison shopping service—which was not a successful service prior to the conduct.^42^

### Conclusions

Ready made abuse categories might not fit new and quickly changing markets. Still, the Google Shopping case demonstrates that the antitrust framework of Article 102 of the TFEU can accommodate a foreclosure theory of harm based on the well established and understood notion of leveraging. The Decision relies on detailed and comprehensive economic evidence which underpins such a foreclosure theory: the evidence on the search habits of users; the visibility and traffic data trends; the correlation of click through rates and ranks; and the critique of Google’s ablation experiment.

## Price Discrimination in State Aid Cases

In 2018, the European Commission adopted two decisions in which the legality of discounts that are granted to large users by public utilities was assessed under state aid rules. These cases identify the circumstances under which subsidies in the form of differential pricing can be considered distortive of competition.

The first case^43^ concerns the change in the structure of waste water charges in Denmark. In particular, Denmark moved from a system of uniform pricing towards a so-called “staircase model”, with three different price levels that depend on the volumes of water that are consumed. The new tariffs thus imply a form of quantity rebate. The case—which started through a complaint—was closed with a finding that the new tariffs were not state aid.^44^

In the second case,^45^ the Commission completed an investigation of rebates of electricity network charges that are granted to large users in Germany. In particular, Germany granted full exemptions from a specific component of network charges to large industrial electricity customers with a steady annual electricity consumption pattern. The Commission concluded that these electricity network charge exemptions constituted illegal state aid and ordered a repayment of the discounts (i.e., a so-called recovery).

While the two cases involved similar issues, they came to different outcomes: one pricing model was cleared with a finding that no state aid was involved, whereas the exemptions from network charges were found to constitute illegal state aid. This illustrates that there are specific circumstances in which differential pricing can be caught by state aid rules.

### Price Discrimination and Distortions of Competition

Price discrimination in the context of state aid control can distort competition. In state aid cases price discrimination typically results from the intervention of the State, for example by applying different tax rates to various groups of companies. The core economic question in such cases is whether such price discrimination can be justified by economic reasons.

Varian (1989) offers an extensive definition of price discrimination: “*The conventional definition is that price discrimination is present when the same commodity is sold at different prices to different consumers. However, this definition fails on two counts: different prices charged to different consumers could simply reflect transportation costs, or similar costs of selling the good; and price discrimination could be present even when all consumers are charged the same price* - *consider the case of a uniform delivered price. We prefer Stigler’s (1987) definition: price discrimination is present when two or more similar goods are sold at prices that are in different ratios to marginal costs.*”^46^ Economic literature thus links price discrimination to price differences that cannot be attributed to differences in costs. If serving different users or groups of users causes different costs, it may be economically reasonable to apply different prices to these users, to reflect marginal cost differences. In such case, differential pricing does not constitute price discrimination in the economic sense.

Beyond differences in marginal costs of serving different users, variations in demand sensitivity may also justify differential prices. In regulated industries—such as electricity or telecommunications—where high fixed costs need to be recovered, Ramsey pricing is typically considered to be an efficient pricing strategy. With Ramsey pricing—a form of third-degree price discrimination—groups of users are charged prices that are inversely related to their demand elasticity, so that price-sensitive customers pay lower prices (and vice versa). Such pricing enables utilities to recover fixed costs while minimizing consumption distortions.

Hence, preferential pricing can constitute price discrimination, though not necessarily, and price discrimination is not necessarily distortive. These two economic principles appear to underpin the application of state aid rules to differential pricing.

### Price Discrimination and State Aid Case Practice

#### The Interplay Between State Aid and Antitrust

Prior to describing briefly relevant past cases, it is worth mentioning that the interplay between antitrust^47^ and state aid rules with respect to price discrimination was the (brief) object of an investigation by the Supreme Court in Latvia.^48^ The case was closed without a judgment as the parties settled; but the questions that were raised are nonetheless interesting and may re-emerge in the future.

In 2016, Latvia’s Supreme Court asked the European Court of Justice whether price discrimination could fall at the same time under state aid rules and antitrust rules, and if so, whether there was a hierarchy between the two sets of rules. The request came in the context of a complaint that discounts that the Riga airport granted to Ryanair constituted illegal state aid. The complaint came after the Riga airport had been fined by the Latvian competition authority for abusing its dominant position when charging high (discriminatory) prices to Air Baltic relative to the lower prices that were granted to Ryanair.

If the discounts are illegal state aid, they should be reimbursed by Ryanair to the Riga airport. Alternatively, if the discounts are deemed no state aid or legal state aid, could the same practice constitute an abuse of dominant position whilst being considered non distortive under state aid rules? In theory, distortions of competition that are caused by discriminatory pricing on a relevant market should be the same whether assessed from an antitrust or a state aid angle. What may differ, however, is the potential for benefits considered relevant in state aid practice to outweigh distortions (e.g., regional development or connectivity objectives). This could mean that abusive price discrimination could in theory be deemed legal state aid, though such an outcome would depend on the balancing done in a compatibility assessment.

#### Differential Pricing in State Aid: Relevant Case Precedents

With regard to relevant state aid precedents that involve differential pricing, such practices have mostly either been deemed the absence of state aid^49^ or illegal state aid. The main sectors that have been covered include energy, aviation, and infrastructure cases.

The fact that differential pricing is no state aid if it can be “*objectively justified by economic reasons*” was decided by the Court of Justice 30 years ago. In case C-67/85, *Kwekerij Gebroeders van der Kooy BV v Commission*,^50^ the Court examined lower tariffs for natural gas used to heat glasshouses of horticultural producers in the Netherlands (i.e., a form of third-degree price discrimination). Whilst the Court sided with the Commission that the lower gas prices to horticultural producers were illegal aid, it opened the possibility to provide justifications for such prices, implicitly based on customer demand elasticities.^51^

Preferential electricity prices that were offered to certain paper mills were found not to constitute state aid in the EDF case.^52^ Based on an economic study and comments by the French authorities, EDF’s pricing was considered in line with marginal cost coverage supplemented with a “*significant proportion*” of its fixed costs, though for some firms it was below total costs. This was deemed to make “*commercial sense*” in the specific context of overcapacity in a market with a lawful monopoly (and paper mills could have installed gas-powered drying equipment as an alternative). A number of electricity cases followed in the late 2000 s/early 2010 s, where discounts to large electricity users were, to the contrary, deemed illegal state aid.^53^

In the Aviation Guidelines,^54^ price differentiation is considered a standard business practice, so long as from an ex-ante perspective the contract between airports and airlines is expected to cover the incremental costs of the airport that serves the airline. The contract should also be “*part of the implementation of an overall strategy of the airport expected to lead to profitability at least in the long term*.”^55^ The incremental cost test thus introduces a minimum threshold for cost coverage that does not determine an arbitrary minimum proportion for contributions to fixed costs. It is implicitly expected that a relevant portion of fixed costs should be covered; but it is up to the airport to determine from which customers and to what extent fixed costs are recovered beyond incremental costs generated by the contractual relationship with a given airline.^56^

Our review reveals some principles for the assessment of preferential pricing in state aid cases: First, prices that cover marginal costs and a significant portion of fixed costs can be considered a reasonable commercial strategy (even if total costs are not covered). Differential prices that are justified by cost differences are thus typically not covered by state aid control. Second, preferential prices that are necessary to avoid customer switching can also be considered justifiable pricing behaviour (this would fit into Ramsey pricing arguments).

If neither cost nor demand-side justifications can be demonstrated, it is likely that differential pricing would be covered by state aid rules and subject to a compatibility assessment (i.e., ascertaining whether benefits outweigh the distortions). Whilst there are findings of illegal aid (e.g., preferential electricity tariffs), there is limited case practice that identifies when price discrimination would constitute compatible state aid. A notable exception is in the area of SGEI where price discrimination can be the *purpose* of a public policy and state aid rules ensure the absence of over-compensation for the cost of serving the obligation.^57^

### Market Conformity of Subsidies in the Form of Rebates: Recent Cases

The principles described above were applied in two recent cases where the Commission investigations focused on the economic rationales that motivated differential tariffs.

#### Case SA.37433—Waste Water Charges, Denmark

The Commission investigated a complaint by the Danish Slaughterers’ Association (*Danske Slagtermestre*), which represented small and medium-sized slaughterhouses in Denmark.^58^ The association complained that illegal state aid was granted to large Danish slaughterhouses through a reduction in waste water charges. Whilst a uniform per m^3^ charge used to be charged for all volumes of waste water, a 2013 law aimed at introducing a more cost-oriented approach: moving from uniform pricing to a so-called “staircase model”.

The new staircase model involves three levels of (decreasing) marginal charges, which depend on water consumption: one price for small volumes [0–500 m^3^/year]; one price for medium volumes [500–20,000 m^3^/year]; and one price for high volumes [above 20,000 m^3^/year]. The m^3^ waste water rate for medium volumes is 20% below the rate for small volumes, and the rate for large volumes is 60% below the rate for small volumes. The rebate is not retroactive: a consumer with waste water demand of 1000 m^3^/year pays the charge that applies for small volumes for the first 500 m^3^, etc. These general pricing principles apply to all consumers in Denmark. As total waste water costs differ across different plants, the pricing scheme results in different levels of waste water charges in the municipalities that are served by these plants.

**Fig. 1:**
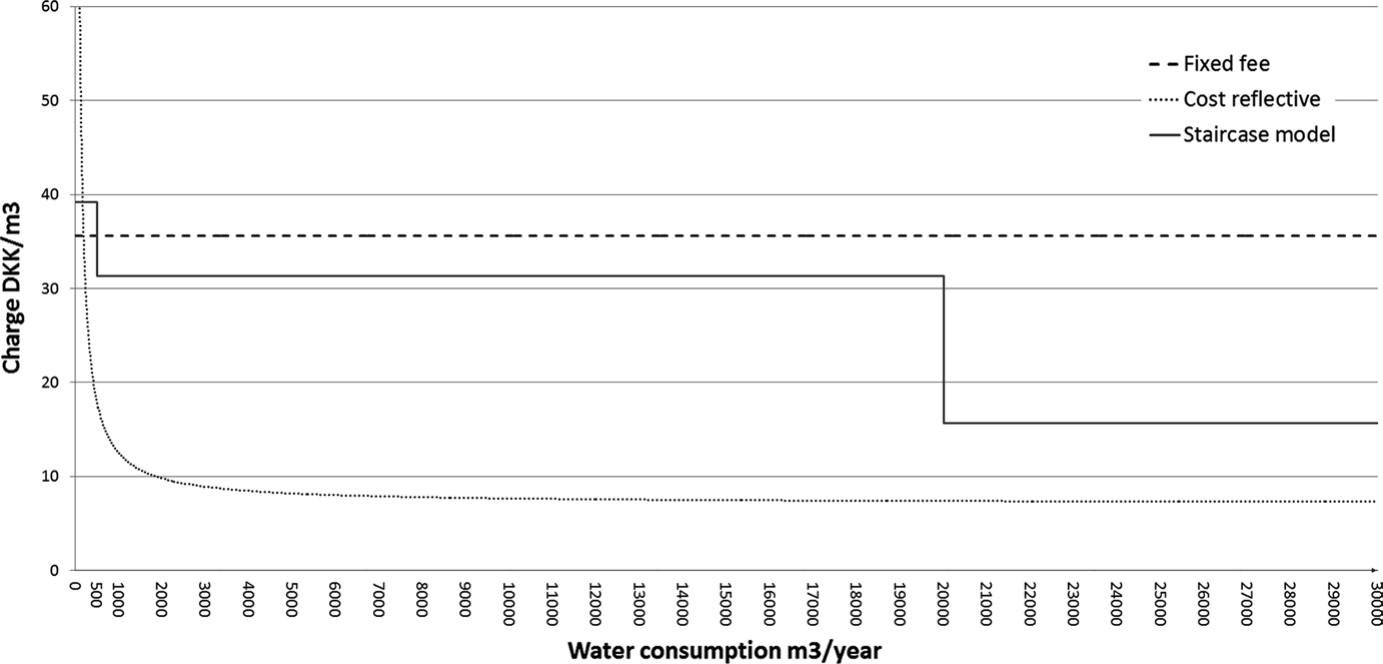
An illustration of incremental costs and the former and new fee systems (Illustration based on Fig. 1 of Commission Decision SA.37433)

The Danish authorities’ stated aim with the new staircase payment model was to align waste water charges with the *costs* that various consumers *cause* to the waste water network. In particular, Denmark argued, also based on an expert report, that approximately 80% of waste water utilities’ costs tend to be fixed over a reasonable planning horizon, while the remaining 20% are variable. It argued that allocating variable costs fully to each consumer and dividing fixed costs equally among connection points is a reasonable approximation of costs caused. As Fig. 1 shows, the “*Cost reflective*” curve decreases steeply as volumes increase. The “*Fixed tax*” line represents the initial uniform fee. Since the total costs of waste water utilities differ by municipality, the staircase method results in different effective fees for these plants.

With an 80%/20% split, most fixed costs are constant over the medium term (irrespective of water consumption). In this case, the method of dividing fixed costs equally by connection point and total variable costs in proportion to consumed quantity would, if anything, lead to an estimate of costs that are *above* truly incremental costs. As Fig. 1 shows, unit prices that are paid by the larger customers cover the estimate of average total unit costs.

The Commission undertook a sensitivity analysis—using actual cost data from a number of Danish waste water utilities—that showed that even if the variable/fixed costs ratio was considerably different (with the share of variable costs increasing substantially), the conclusion would still remain that the newly discounted prices for larger customers are higher than the costs that these customers are estimated to cause.

Beyond the cost evidence, the Commission also found that the observed tariffs were consistent with a form of Ramsey pricing, whereby larger customers may have more elastic demand than smaller ones. The Commission took into account evidence that demonstrated that larger waste water customers could credibly opt-out from the waste water system by building their own treatment facilities that are not connected to the main sewage system.^59^ Potential inefficiencies that arise from highly decentralised waste water treatment is recognized in the literature on the economics of the water sector and in a recent report of the European Environmental Agency on water pricing.^60^

#### Case SA.34045—Electricity Network Charges, Germany

In 2011, Germany granted large industrial electricity customers a full exemption from their electricity network charges if their annual electricity consumption reached both 7000 annual hours of full usage and 10 GWh of energy. The Commission’s investigation focused on the period 2011–2013 (after which a new tariff system was introduced that eliminated the full exemptions). Questioning the economic justification of a full exemption, the Commission opened a state aid investigation procedure in 2013. The investigation was triggered by complaints from customer associations, utility companies, and citizens that alleged that the exemption for large and steady electricity users constituted illegal state aid.

Germany justified the full exemptions from network charges on grounds of cost-causation. In particular, Germany argued that balancing costs for sudden variations in the demand are not caused by baseload consumers, as they have a predictable and constant consumption pattern. This reduced the need to keep reserves. The same applies for the costs that are caused by measures that are necessary to keep the frequency of the network stable despite variations in load. Finally, Germany also invoked an argument that reflected a Ramsey pricing logic by claiming that the annual energy consumption threshold of 10 GWh was necessary to qualify for the exemptions, because for large consumers opting out of the electricity network by connecting directly to power plants is a realistic alternative.

The Commission recognised that cost causality is a relevant principle to assess whether the pricing schedule could be justified on economic grounds. The Commission also recognized that the arguments that were advanced by Germany may have justified (between 2011 and 2013) some reductions from the generally applicable network charges to large consumers with a steady load profile. The methodology of setting general network charges in Germany did not take into account that steady load profiles may imply lower overall costs for the network as a whole.

However, given that even large consumers with steady load profile caused *some* network costs, the Commission considered that a *full exemption* from paying the charges cannot be justified and ordered the recovery of the granted state aid. In order to determine the amount to be recovered (i.e., the “market price” that was not paid), the Commission did not order a calculation of actual costs caused by the consumers concerned. Instead, it accepted that the amount to be recovered would be determined using the methodology that was applicable before 2011 to determine individual network charges of baseload consumers.^61^ The Commission considered that methodology as a reasonable proxy of individual network charges during 2011–2013.

### Conclusions

As is illustrated by the waste water case in Denmark and the network charges case in Germany, economic principles underpin the assessment of state measures that potentially involving illegal subsidies in Europe.

In order to determine whether differential pricing is at all covered by state aid rules, it has to be assessed whether lower prices that are paid to public undertakings (or reduced through public intervention) can be justified by lower costs of production. Whilst full cost coverage is not necessary, pricing needs to be related to the costs of serving specific firms or groups of firms (e.g., at least variable costs plus a portion of fixed costs or incremental costs). Demand-side considerations and optimal fixed cost recovery are equally relevant. Differential pricing may constitute illegal state aid if it leads to price discrimination that cannot be justified by commercial strategies that are consistent with optimal pricing decisions and if it does not generate economic benefits outweighing competition distortions.

For state aid control, the concern is that specific firms or groups of firms benefit—through public funds—from preferential tariffs relative to their competitors. More favourable prices that are not justified either by cost differences or by efficient pricing strategies may be distortive if they artificially reduce the price of inputs for beneficiaries of aid (or increase relative input prices for rivals). This could lead to competition distortions unless justified by other objectives with positive welfare effects (e.g., regional development, or social objectives).

## Counterfactual Analysis in Recent Mergers

Counterfactual analysis is at the core of effects-based competition assessments. In merger control, it involves comparing whether a merger is likely to restrict competition relative to how the market would have evolved absent the proposed transaction. In many instances, this means comparing the expected post-merger equilibrium with the observed pre-merger market conditions. The analysis becomes more complex when significant changes are anticipated even in the absence of the proposed transaction: when the forward-looking counterfactual to the merger differs from the pre-merger equilibrium.

Such a change in market circumstances in the absence of the merger can be caused, for instance, by expected entry or exit or by foreseeable shifts in market demand.^62^ In what follows, we denote such situations as “non-standard counterfactuals”, since they differ from the status quo.

Cases with non-standard counterfactuals often involve complex economic assessments. In particular, they require making a prediction not only about the competitive outcome with the proposed transaction, but also without it. In this section, we discuss a number of recent EU mergers in which the analysis of such counterfactuals played a central role. Specifically, we discuss the economic principles that were applied when assessing the competitive effects of mergers where the counterfactual deviated from the status quo.

### Recent Cases Involving Counterfactual Analysis

In 2017, three transactions were notified to the Commission that raised complex questions about the nature of the competitive counterfactual:

- *Knorr*-*Bremse*/*Haldex* concerned the proposed acquisition of Haldex by Knorr-Bremse (two of world’s largest manufacturers of brake systems and components for trucks)^63^;
- *Lufthansa*/*Air Berlin* concerned the attempted takeover by Lufthansa of assets coming out of the insolvency of rival airline Air Berlin (in particular, planes and takeoff/landing slots)^64^; and
- *ArcelorMittal*/*Ilva* concerned the takeover of financially distressed steel producer Ilva by rival ArcelorMittal.^65^

In *Knorr*-*Bremse*/*Haldex*, Knorr-Bremse’s last minute offer for Haldex had outbid a prior takeover offer by automotive supplier ZF Friedrichshafen, which had already secured antitrust clearance from all relevant authorities. ZF had been keen to acquire Haldex’s braking expertise to complement its own autonomous driving technology. It had announced the intention to make significant investments to expand Haldex’s activities, effectively trying to turn it from a niche operator into a full-service provider that would compete more effectively with market leaders Knorr-Bremse and Wabco.^66^ Eventually, that transaction did not take place, since Knorr-Bremse pre-empted it through a higher bid.

From an economic perspective, the Commission therefore had to assess whether Knorr-Bremse’s takeover proposal had the object or effect of blocking potentially pro-competitive entry by ZF into the brake market. More specifically, it had to consider whether the correct counterfactual to the transaction was the status quo ante or the counterfactual of a takeover of Haldex by ZF Friedrichshafen. Accordingly, a key element of the assessment was the content of Knorr-Bremse’s internal documents and business plans, to obtain a realistic perspective of expected industry evolutions, commercial motivations, and likely competitive consequences.

In *Lufthansa/Air Berlin*, the proposed merger of airlines gave rise to an unprecedented number of 130 overlap routes (with around 70 of them becoming monopoly or near-monopoly routes), in addition to increased airport dominance at Düsseldorf airport. Even so, Lufthansa argued that the proposed acquisition would bring about no restriction of competition, because there were no viable alternative bidders for Air Berlin’s struggling assets. Compared to the non-standard counterfactual advanced by Lufthansa, the transaction was therefore innocuous.

Yet, for a substantial part of Air Berlin’s former operations (specifically, the Austrian subsidiary NIKI), the Commission contested the view that these assets would not have been commercially viable absent a takeover by Lufthansa. In particular, several other carriers had made offers in the public takeover process for NIKI, but those bids had been discarded by the insolvency administrator in favor of initiating exclusive negotiations with Lufthansa.^67^ As in *Knorr*-*Bremse/Haldex*, the Commission therefore had to determine whether an alternative takeover of the target company was a more appropriate counterfactual against which the proposed transaction should be assessed. After all, it would prima facie not appear implausible that a strong incumbent airline might have an incentive to block entry of a competitor by acquiring a struggling rival instead of allowing that competitor to acquire the struggling firm.

Finally, the Italian steel maker Ilva, which runs the largest integrated steel mill in Europe, had entered into insolvency proceedings in 2015 and was being sold through a competitive tender. In *ArcelorMittal/Ilva* the Commission therefore had to assess whether or not there would have been a viable alternative purchaser for Ilva in the absence of the acquisition by ArcelorMittal. In the case at hand, a parallel state aid investigation had found that there were indeed such viable alternatives.^68^ Even so, the case involved thorny questions on the degree to which these alternatives would have materialized and would have been comparable, since they involved different business models and planned approaches toward a necessary restructuring.

### Principles of Counterfactual Analysis

There is a widespread (if inaccurate) perception that is sometimes also promoted by merging parties that merger control intrinsically involves a comparison of pre- and post-merger situations. In reality, merger assessments always compare the expected future situation with and without a merger.^69^ Merger control, thus, is generally forward-looking, as it compares two unknown future outcomes.

Within this framework, the pre-merger situation is of importance, in so far as it is an informative predictor of how the market would look in the absence of the proposed transaction. Very often, this is a reasonable assumption, which makes the status quo a sensible counterfactual.

In other situations, however—particularly in markets that are undergoing material changes—it is not reasonable to assume that the future will look like the past.^70^ Possible reasons for a foreseeable divergence of future market conditions from the status quo—and, thus, for employing a non-standard counterfactual—can for instance include the following: a structural decline of demand, which will lead to overcapacities in the future; a failing firm defense; the impending entry or exit of a significant potential rival; or the likely ascent of a smaller competitor that will grow into an important competitive force.

These examples show that non-standard counterfactuals can make mergers either more or less problematic than a comparison to the status quo ante. E.g., in *Knorr*-*Bremse/Haldex*, the existence of an alternative bidder with complementary assets and fewer competitive overlaps made the proposed transactions more problematic than would otherwise have been the case. Conversely, in the non-NIKI part of *Lufthansa/Air Berlin* the diminishing standalone viability of the respective targets made the proposed transaction less problematic than would otherwise have been the case. Finally, in *ArcelorMittal/Ilva* and in the NIKI part of *Lufthansa/Air Berlin,* the Commission maintained the pre-merger situation as the relevant counterfactual and rejected the notion that the targets would continue to decline absent the transaction because of the existence of alternative viable purchasers at the time of the tender awards.

While merger counterfactuals may deviate substantially from the pre-merger situation, it is also clear that such scenarios cannot simply be conjured based on speculative possibilities. Instead, credible evidence has to be produced if either the Commission or merging parties propose to deviate from the pre-merger situation as the appropriate counterfactual.

Arguably, there is a sliding scale in this regard that depends on the degree of deviation from the pre-merger scenario.^71^ This precludes the Commission from judging a proposed merger against arbitrary alternative combinations that involve one of the parties with potentially better competitive outcomes. When reliable evidence exists that a specific alternative transaction is the most plausible alternative course of events, however, it would make little sense to evaluate the proposed merger against a status quo ante that may no longer be relevant for the future or against other counterfactuals that ignore the existence of the alternative transaction. For instance, in a previous case from 2009 (*Lufthansa/Austrian Airlines*), the Commission found that—absent the proposed transaction—Austrian would likely have been acquired by Air France-KLM, which made the latter a reasonable counterfactual.^72^

An important question when applying non-standard counterfactuals, however, is what precisely is meant by considering market outcomes “absent the proposed transaction”. In particular, it can make a large difference whether one means “absent the merger proposal” (the ex-ante perspective) or “absent the clearance and implementation of the proposed merger” (the ex-post perspective). For instance, in all three of the recent cases the issue arose whether the rival bidders for the respective targets would still have an interest in acquiring them at the time of the assessment by the Commission, since their bids formally proved interest only at the time of their respective offers (which was several months prior to the eventual merger notifications).

A potential divergence between the ex-ante and ex-post approach towards counterfactuals can pose complex trade-offs between deterrence and efficiency considerations. For instance, consider the situation where the interest of an alternative purchaser has become less certain ex-post due to a deterioration of the ailing target’s commercial situation. Pursuing the ex-post approach may then imply having to permit acquisitions that have a negative effect on competition relative to the situation where that transaction had not been proposed. This may go as far as having to clear transactions with demonstrably exclusionary intent, whose purpose is to prevent other purchasers from entering the market.^73^ Conversely, pursuing the ex-ante approach may imply having to block a proposed merger even though the applied counterfactual will not in fact materialize.

While it may be tempting to focus on what is achievable at the time of the decision, such an approach may allow merging firms to obtain clearance for a merger by presenting authorities with the *fait accompli* that the ex-ante counterfactual is no longer available as a result of the proposed deal itself. Using an ex-ante perspective can deter such hold-up strategies. However, it may not always lead to the ex-post optimal outcome.

In our view, the ex-ante approach is a more sensible policy approach, since it is directed at preventing anticompetitive outcomes at their root. Arguably, it also comes closest in spirit to the stated aim of comparing “the competitive conditions that would result from the notified merger with the conditions that would have prevailed without the merger.”^74^

It is important to realize that the ex-ante approach does not ignore observable developments post-announcement of the transaction. Rather, post-notification developments that are unrelated to the merger are taken into account in both the merger scenario and the counterfactual. However, post-notification developments that are causally related to the transaction can only be attributed to the merger scenario. For instance, in *GE/Alstom*, the Commission found that a “recent deterioration of Alstom’s financial situation in so far as it would not have occurred in the absence of the proposed merger cannot be taken into account”.^75^ The post-notification decline of the target in that case was therefore causally attributed to the merger.

### Case Application

Eventually, none of the proposed transactions proceeded as originally notified by the parties. Both Knorr-Bremse and Lufthansa ultimately decided to withdraw their respective takeover bids for Haldex and NIKI.^76^ *ArcelorMittal/Ilva*, on the other hand, was cleared in the second phase, subject to extensive divestments that consisted of integrated steelworks and finishing lines in five different EEA countries (Italy, Romania, the Czech Republic, Belgium, and Luxembourg).

Since the non-merger development is ex-post observable when a transaction ultimately does not proceed, it is interesting to compare the real-world outcomes following those cases with the counterfactual predictions that were put forward during the merger control process. For *Lufthansa/Air Berlin*, the contested NIKI part was ultimately purchased by rival Ryanair (via Laudamotion). Arguably, this outcome is even more competitive than the pre-merger situation, since Ryanair is an aggressively competing low cost carrier. The appropriate use of counterfactual therefore appears to have prevented material competitive damage in this case, which may have arisen if Lufthansa had acquired the target.

Conversely, the withdrawal of *Knorr*-*Bremse/Haldex* did not lead the alternative bidder ZF Friedrichshafen to seize the opportunity and proceed with its earlier intention of taking over Haldex. It has been reported that the main reason for this change of heart was that ZF’s supervisory board became increasingly concerned about management’s costly M&A aspirations (which included an abandoned takeover attempt of Knorr-Bremse’s other competitor, Wabco, subsequent to the failed Haldex bid).^77^

This divergence of ex-ante and ex-post outcomes illustrates the potential challenges that are associated with non-standard counterfactuals. First, considerations of ex-ante deterrence do not necessarily coincide with ex-post efficiency in terms of competitive outcomes. Nevertheless, as discussed above, we believe that prioritizing incentive effects through an ex-ante approach is a more sensible policy.

Second, if the intent of a merger proposal is to block the entry of a rival, the purchaser may succeed with its aim even if the acquisition proposal is ultimately prohibited as alternative bidders may have lost its interest in the target during the merger proceedings. In practice, the latter case is not an unlikely outcome, since the length of takeover proceedings may well imply further deterioration of the commercial viability of ailing targets. In certain cases, deterrence of anticompetitive takeover attempts may therefore require considering additional antitrust instruments (such as Article 102) to be fully incentive-compatible.

## Conclusion

2017-2018 was again an interesting and challenging period for DG Competition and the CET, both by the mere number of cases to be assessed as well as the variety of topics that were raised across instruments. Moreover, given the pipeline of appeals, the European Courts will review some important issues and cases in the future, including in particular the Commission’s assessment of pay-for-delay conduct (Servier, Lundbeck), exclusivity conduct (Intel), Qualcomm (exclusivity payments), and a merger case in mobile telephony (Hutchison 3G UK/Telefonica UK). Moreover, a number of debates, such as innovation and merger control, or the impact of rising profit margins, are still very lively. The coming years thus promise to be interesting.

## Footnotes

1 A detailed overview of DG Competition’s activity can be found in its Annual Activity Report. The report also illustrates how DG Competition enforced the competition rules of the European Union in 2017. The 2017 Annual Activity Report is available at https://ec.europa.eu/info/publications/annual-activity-report-2017-competition_en.

2 Case AT.40153. See DG Competition’s press release available at http://europa.eu/rapid/press-release_IP-17-1223_en.htm. See also Sect. 3 of Buehler et al. (2017) for an in-depth assessment of the CET’s work in this case.

3 Case AT.39740. See DG Competition’s press release available at http://europa.eu/rapid/press-release_MEMO-17-1785_en.htm.

4 Case AT.39813. See DG Competition’s press release available at http://europa.eu/rapid/press-release_IP-17-3622_en.htm. The Commission fined Lithuanian Railways (Lietuvos geležinkeliai) an amount of EUR 27.87 million for hindering competition in the rail freight market, in breach of European Union antitrust rules, by removing a rail track that connects Lithuania and Latvia.

5 Case AT.40208. See DG Competition’s press release available at http://europa.eu/rapid/press-release_IP-17-5184_en.htm. The Commission decided that International Skating Union (ISU) rules that impose severe penalties on athletes who participate in speed skating competitions that are not authorised by the ISU are in breach of European Union antitrust law. The ISU must now change these rules.

6 Case AT.40220. See DG Competition’s press release available at http://europa.eu/rapid/press-release_IP-18-421_en.htm. The Commission fined Qualcomm EUR 997 million for abusing its market dominance in LTE baseband chipsets. Qualcomm prevented rivals from competing in the market by making significant payments to a key customer on condition it would not buy from rivals. This is illegal under European Union antitrust rules.

7 Case AT.38700. See DG Competition’s press release available at http://europa.eu/rapid/press-release_IP-18-3401_en.htm. The Commission found that Greece had infringed competition rules by giving the state-owned electricity incumbent, PPC, privileged access rights to lignite, and called on Greece to propose measures to correct the anti-competitive effects of that infringement. Due to appeals at both the European General Court and European Court of Justice, such corrective measures had not been implemented and the decision concludes that the amended final version of the remedies submitted by Greece fully addresses the infringement identified by the Commission.

8 Case AT.39816. See DG Competition’s press release available at http://europa.eu/rapid/press-release_IP-18-3921_en.htm. The Commission imposed binding obligations on Gazprom to enable the free flow of gas at competitive prices in Central and Eastern European gas markets, addressing the European Commission’s preliminary concerns that Gazprom breached EU antitrust rules by pursuing an overall strategy to partition gas markets along national borders in eight Member States (Bulgaria, the Czech Republic, Estonia, Hungary, Latvia, Lithuania, Poland, and Slovakia), which may have enabled Gazprom to charge higher gas prices in five of these Member States (Bulgaria, Estonia, Latvia, Lithuania, and Poland).

9 Cases AT.40465 (Asus), AT.40469 (Denon & Marantz), AT.40181 (Philips) and AT.40182 (Pioneer). See DG Competition’s press release available at http://europa.eu/rapid/press-release_IP-18-4601_en.htm. The Commission fined these companies an overall amount of EUR 111 million for restricting the ability of their online retailers to set their own retail prices for widely used consumer electronics products, such as kitchen appliances, computer notebooks, and hi-fi products.

10 Case AT.40099. See DG Competition’s press release available at http://europa.eu/rapid/press-release_IP-18-4581_en.htm. The Commission fined Google EUR 4.34 billion for imposing illegal restrictions on Android device manufacturers and mobile network operators to cement its dominant position in general internet search.

11 Case AT.37990; Case T-286/09—Intel v Commission before the General Court; and Case C-413/14 P—Intel v Commission before the Court of Justice, ECLI:EU:C:2017:632.

12 Case C-177/16—Biedrība “Autortiesību un komunicēšanās konsultāciju aģentūra—Latvijas Autoru apvienība” Konkurences padome, judgment of the Court of Justice of 14 September 2017, ECLI:EU:C:2017:689. See also European Commission (2018, p. 7).

13 Detailed statistics on the number of merger notifications and decisions are available at http://ec.europa.eu/competition/mergers/statistics.pdf.

14 A simplified procedure is used for proposed transactions that by their nature do not raise competition concerns and do not normally require a market investigation (for example, when there is no horizontal overlap and no vertical relationship between the merging parties). See Commission Notice on a simplified procedure for treatment of certain concentrations under Council Regulation (EC) No 139/2004, OJ C 366 of 14.12.2013, page 5.

15 Details on the European Union merger regulation are available at http://ec.europa.eu/competition/mergers/procedures_en.html.

16 The transactions that have been authorized subject to remedies are: M.7932—Dow/DuPont (March 2017); M.7962—ChemChina/Syngenta (April 2017); M.8306—Qualcomm/NXP semiconductors (January 2018); M.8084—Bayer/Monsanto (March 2018); M.8444—ArcelorMittal/Ilva (May 2018); and M.8451—Tronox/Cristal (July 2018).

17 The transaction that has been authorized without remedies is: M.8394—Essilor/Luxottica (March 2018).

18 The transactions that have been prohibited are: Case M.7995—Deutsche Börse/London Stock Exchange Group (March 2017); and Case M.7878—HeidelbergCement/Schwenk/Cemex Hungary/Cemex Croatia (April 2017). The transactions that have been abandoned during the in-depth investigation are: Case M.7095—SOCAR/DESFA (notified in October 2014, withdrawn in February 2017); Case M.8222—Knorr-Bremse/Haldex (notified in June 2017 and withdrawn in September 2017); and Case M.8547—Celanese/Blackstone/JV (notified in September 2017 and withdrawn in March 2018).

19 For the 12-month period from January to December 2017, the corresponding statistics are: 380 merger notifications amongst which 280 simplified cases, 55 clearances in first-phase without remedies and 18 with remedies, 2 clearances in second-phase with remedies, 2 prohibitions and 2 withdrawals during the in-depth investigation.

20 See, for example, Sect. 2 of Buehler et al. (2017).

21 See in particular Annex 5 to the decision in case M.7932—Dow/DuPont, available at http://ec.europa.eu/competition/elojade/isef/case_details.cfm?proc_code=2_M_7932.

22 Case T-194/13—United Parcel Service v Commission, judgment of the General Court of 7 March 2017.

23 Case C-265/17 P—Commission v United Parcel Service before the Court of Justice.

24 Detailed statistics related to the Commission’s State Aid activity are available at http://ec.europa.eu/competition/publications/annual_report/2017/part2_en.pdf.

25 Case SA.38373. See DG Competition’s press release available at http://europa.eu/rapid/press-release_IP-16-2923_en.htm.

26 Case SA.38373. See DG Competition’s press release available at http://europa.eu/rapid/press-release_IP-16-2923_en.htm.

27 Case SA.44888. See DG Competition’s press release available at http://europa.eu/rapid/press-release_IP-18-4228_en.htm.

28 Capacity mechanims are measures put in place by Member States to ensure sufficient investment in energy capacity.

29 Judgment of the General Court of the EU of 25 January 2018 in Case T-818/14. The CET had contributed to determining the market conform concession fee in the decision that was upheld.

30 Judgment of the General Court (Fifth Chamber) of 12 July 2018 in Case T-356/15.

31 Feed-in-tariffs are guaranteed fixed prices offered on a temporary basis to incentivize investment in energy capacity that would not otherwise occur.

32 The abuse took place in all 13 EEA Member States where Google rolled out a comparison shopping product. The starting dates of the abuse varied by country affected from 2008 to 2013.

33 See Prufer and Schottmüller (2017).

34 See for instance Rochet and Tirole (2006).

35 Commission decision of 27 June 2017 relating to a proceeding under Article 102 of the Treaty on the Functioning of the European Union and Article 54 of the EEA Agreement (Case AT.39740—Google Search (Shopping)), Sect. 7.2.3.1.

36 European Commission (2009, para. 19).

37 Case AT.39740—Google Search (Shopping) Article 7 Regulation (EC) 1/2003, page 42.

38 Case AT.39740—Google Search (Shopping) Article 7 Regulation (EC) 1/2003, page 35. See also https://www.blog.google/around-the-globe/google-europe/european-commission-decision-shopping-google-story/.

39 Also pointed out in Fumagalli et al. (2018, p. 605).

40 The introduction of the “Panda” change to the Google search ranking algorithm ranked lower websites with 'non-original' content.

41 The weekly Visibility Index of Sistrix measures the changes in the Google Search ranking of individual sites, based on a large number of keywords. The keywords are chosen in such a way that they reflect the average search behaviour for each country.

42 Case AT.39740—Google Search (Shopping) Article 7 Regulation (EC) 1/2003, page140, recital 490.

43 SA.37433, Alleged State Aid through discounts on waste water charges, available at: http://ec.europa.eu/competition/elojade/isef/case_details.cfm?proc_code=3_SA_37433.

44 The Commission decision is currently under appeal before the General Court (Case T-486/18).

45 SA.34045, Exemption from network charges for large electricity consumers in Germany, available at http://ec.europa.eu/competition/elojade/isef/case_details.cfm?proc_code=3_SA_34045.

46 Varian (1989, p. 598).

47 With respect to antitrust, the ECJ has recently clarified in the MEO Judgment regarding price discrimination under Article 102 c) that “*the mere presence of an immediate disadvantage affecting operators who were charged more, compared with the tariffs applied to their competitors for an equivalent service*” is not sufficient for a finding of abuse (paragraph 26). But rather, the relevant test is whether “*the behaviour of the undertaking in a dominant position tends, having regard to the whole of the circumstances of the case, to lead to a distortion of competition between […] business partners*” (paragraph 27). The case reference is C-525/16.

48 e C-159/16: http://curia.europa.eu/juris/liste.jsf?language=fr&num=C-159/16.

49 The test of whether rebates are covered by state aid control depends on whether the subsidies in form of rebates are attributable to the State, if they are selective (i.e., only offered to some firms), if they provide an advantage (i.e., are not in line with what a market operator would decide to charge) and if they are liable to distort competition and affect trade between Member States.

50 The judgment is available at: https://eur-lex.europa.eu/legal-content/GA/TXT/?uri=CELEX:61985CJ0067.

51 See paragraph 2 of the summary of the Judgment that indicates that a tariff could be justified if set so as: “*to resist competition on the same market from other sources of energy the price of which was competitive. In determining whether such competition is a real prospect account should be taken not only of the different price levels but also of the costs involved in conversion to a new source of energy, such as replacement and depreciation costs for heating equipment.*” In other words, retaining price-sensitive customers (with real alternatives and low costs of switching) can justify lower prices; but this was not complied with in this case, based on studies that showed that the prices that were charged were lower than necessary to avoid conversion to coal.

52 Commission Decision 2001/274/EC of 11 April 2000 (*EDF)* EE [2001] L 95/18: “*In principle, a private or public enterprise can charge different prices according to the use made of the products it sells; however, such differences must be justified on commercial grounds, i.e. they must cover the marginal cost and at least a proportion of the fixed costs*” (paragraph 69). See also Paragraphs 70–75.

53 For instance, in its 2011 investigation of lower electricity tariffs that were granted in 2007-2008 by the Greek state-owned Public Power Corporation (PPC) to Aluminium of Greece, the illegal state aid amount was quantified as the difference between the reduced and the ‘standard’ tariff for large industrial consumers. Commission Decision of 13 July 2011 on Case No SA.26117—C 2/10 (ex NN 62/09) implemented by Greece in favour of Aluminium of Greece SA (notified under document C(2011) 4916).

54 See Sect. 3.5 of the Aviation Guidelines https://eur-lex.europa.eu/legal-content/EN/TXT/?uri=CELEX%3A52014XC0404%2801%29.

55 See paragraph 66 of the Aviation Guidelines.

56 The approach was confirmed by the Court in Case T-375/15 (*Germanwings vs. Commission*). The Judgment annulled the Commission finding that the contract between Zweibrucken airport and Germanwings contained illegal state aid -- but on grounds that the *evidence* used in the Decision was insufficient to show the *causal* relation between investment costs and Germanwings' presence in the airport. The Court found that it could not be established that expansions of the terminal and hiring of new staff were attributable to Germanwings (and thus should have been covered by the airport charges it paid). The incremental nature of investment costs thus requires detailed evidence of a causal link between the decision to undertake specific investments and a particular contract.

57 In 2014, the Commission concluded that the compensation of a PSO that consisted of a 15% discount off bus fares for children and students in Land Rhineland-Palatinate was compatible state aid (i.e. it was a potentially distortive subsidy but its benefits outweighed the distortions). See: http://ec.europa.eu/competition/state_aid/cases/249123/249123_1526826_204_2.pdf. In this case, price discrimination was the *purpose* of the measure. According to a commentator, the Decision assumed rather than demonstrated that bus companies would not offer the same advantageous prices to students that are more price-sensitive (at least not to the same extent as required by the PSO). The Decision rather focused on the method for calculating the appropriate compensation. See http://stateaidhub.eu/blogs/stateaiduncovered/post/17.

58 See Commission Decision SA.37433 and related Court Case T-486/18.

59 Paragraph 44 of the Decision, SA.37433.

60 EEA (European Environment Agency) “*Assessment of cost recovery through water pricing*.” Technical Report, No. 16/2013, page 22: *“one should be aware of the fact that some users of water services have alternatives available, which may become attractive if the price of the water service becomes too high.[…] in the case of wastewater treatment, they may start building and operating their own private treatment plants. This not only affects the rate of cost recovery for the public (collective) water service investments, but it may also lead to a less efficient use of water resources. Clearly, prices for water services may not only be too low, but also too high to be called 'adequate incentives'.”*

61 The pre-2011 system of network charges that were determined by the so-called *physical path method* did not entail state resources and therefore did not constitute state aid.

62 A proposed merger may in fact be a response to the anticipation of evolving market conditions: e.g., to safeguard the viability of a struggling target or to stave off entry by potential competitors.

63 Case M.8222—Knorr-Bremse/Haldex (notification withdrawn, 4 October 2017).

64 Case M.8633—Lufthansa/Certain Air Berlin Assets (Commission decision of 21 December 2017).

65 Case M.8444—ArcelorMittal/Ilva (Commission decision of 7 May 2018).

66 As Haldex's management explained, “*ZF’s strong capabilities within electronics and software development as well as global reach and customer access offer an excellent opportunity to further develop Haldex, thereby allowing Haldex to continue its development of future braking systems and expansion of its current product portfolio.*” ZF Friedrichshafen press release of 4 August 2016, available at: https://press.zf.com/site/press/en_de/microsites/press/list/release/release_24576.html.

67 “*Air Berlin's insolvency administrator received a number of bids for various parts of Air Berlin. On 25 September 2017, the insolvency administrator announced their decision to continue negotiations exclusively with Lufthansa, Germany's largest airline, for most of Air Berlin's assets, including LGW and NIKI. They thereby rejected the other bids (except for easyJet's, where negotiations continued separately for Air Berlin's Berlin Tegel operations).*” Commission press release of 17 December 2017, available at: http://europa.eu/rapid/press-release_IP-17-5402_en.htm.

68 “*[I]n the right hands, ILVA has a sustainable future. As the sales process run by the Italian government showed, there were several potential bidders ready to invest in ILVA's future and upgrade the site in line with environmental standards.*” Commission press release of 21 December 2017, available at: http://europa.eu/rapid/press-release_IP-17-5401_en.htm.

69 “*In assessing the competitive effects of a merger, the Commission compares the competitive conditions that would result from the notified merger with the conditions that would have prevailed without the merger*.” European Commission (2004, para. 9, footnote omitted).

70 “*In most cases the competitive conditions existing at the time of the merger constitute the relevant comparison for evaluating the effects of a merger. However, in some circumstances, the Commission may take into account future changes to the market that can reasonably be predicted.*” European Commission (2004, para. 9, footnote omitted).

71 For example, there is a high evidentiary standard for accepting a failing firm defence which is an extreme departure from the pre-merger scenario as counterfactual. See European Commission (2004, Section VIII).

72 “*[I]t appears likely that, absent the acquisition by LH, OS would have been acquired by another airline, namely Air France-KLM. Therefore, the most likely foreseeable alternative counterfactual to the pre-merger situation for the assessment of the transaction, would be a foreseeable situation whereby OS is acquired by another airline, more specifically by Air France-KLM.*” Case M.5440—Lufthansa/Austrian Airlines (Commission decision of 28 August 2009, paragraph 85).

73 A monopolist is usually willing to pay more for a strategic asset than will an entrant, because the former is protecting its larger profits, whereas the latter can expect only its share of duopoly profits. This insight was developed (at least as early as) by Gilbert and Newbery (1982).

74 Supra footnote 69.

75 Case M.7278—General Electric/Alstom (Commission decision of 8 September 2015, Sect. 8.10.3.6).

76 Lufthansa proceeded with its takeover of other Air Berlin assets, however, which was cleared subject to remedies at the end of a phase I investigation. See Case M.8633—Lufthansa/Certain Air Berlin Assets (Commission decision of 21 December 2017). Knorr-Bremse stated that its decision to abandon its merger (during the Commission's phase II investigation) was primarily caused by Haldex’ management’s unwillingness to support the process of achieving merger control clearance. See https://www.handelsblatt.com/unternehmen/industrie/autozulieferer-knorr-bremse-gibt-sich-bei-haldex-geschlagen/20345926.html?ticket=ST-986861-DoAKkuaRe0NUpd5jnSou-ap4.

77 E.g., see https://www.suedkurier.de/ueberregional/wirtschaft/Wabco-UEbernahme-ZF-bremst-ZF-aus;art416,9348818.
